# Role of Klotho in Hyperglycemia: Its Levels and Effects on Fibroblast Growth Factor Receptors, Glycolysis, and Glomerular Filtration

**DOI:** 10.3390/ijms22157867

**Published:** 2021-07-23

**Authors:** Marlena Typiak, Tomasz Kulesza, Patrycja Rachubik, Dorota Rogacka, Irena Audzeyenka, Stefan Angielski, Moin A. Saleem, Agnieszka Piwkowska

**Affiliations:** 1 Laboratory of Molecular and Cellular Nephrology, Mossakowski Medical Research Institute, Polish Academy of Sciences, Wita Stwosza 63, 80-308 Gdansk, Poland; tkulesza@imdik.pan.pl (T.K.); prachubik@imdik.pan.pl (P.R.); drogacka@imdik.pan.pl (D.R.); iaudzeyenka@imdik.pan.pl (I.A.); angielsk@gumed.edu.pl (S.A.); apiwkowska@imdik.pan.pl (A.P.); 2 Faculty of Chemistry, University of Gdansk, Wita Stwosza 63, 80-308 Gdansk, Poland; 3 Bristol Renal, Dorothy Hodgkin Building, University of Bristol, Bristol BS1 3NY, UK; M.Saleem@bristol.ac.uk

**Keywords:** Klotho protein, diabetes mellitus, diabetic nephropathy, hyperglycemia, fibroblast growth factor receptors

## Abstract

Hyperglycemic conditions (HG), at early stages of diabetic nephropathy (DN), cause a decrease in podocyte numbers and an aberration of their function as key cells for glomerular plasma filtration. Klotho protein was shown to overcome some negative effects of hyperglycemia. Klotho is also a coreceptor for fibroblast growth factor receptors (FGFRs), the signaling of which, together with a proper rate of glycolysis in podocytes, is needed for a proper function of the glomerular filtration barrier. Therefore, we measured levels of Klotho in renal tissue, serum, and urine shortly after DN induction. We investigated whether it influences levels of FGFRs, rates of glycolysis in podocytes, and albumin permeability. During hyperglycemia, the level of membrane-bound Klotho in renal tissue decreased, with an increase in the shedding of soluble Klotho, its higher presence in serum, and lower urinary excretion. The addition of Klotho increased FGFR levels, especially FGFR1/FGFR2, after their HG-induced decrease. Klotho also increased levels of glycolytic parameters of podocytes, and decreased podocytic and glomerular albumin permeability in HG. Thus, we found that the decrease in the urinary excretion of Klotho might be an early biomarker of DN and that Klotho administration may have several beneficial effects on renal function in DN.

## 1. Introduction

The selective filtration of plasma is performed in the cortex of the kidney in the glomerulus of the nephron. Endothelial cells of blood arteries that enter the glomerulus, a glomerular basement membrane (GBM) between them and podocytes, and podocytes themselves form the glomerular filtration barrier (GFB). Podocytes are highly specialized cells that are dedicated to plasma filtration. They consist of a cell body and major and foot processes. Foot processes branch out from major processes and tightly cover blood arteries in the glomerulus. Between neighboring foot processes, a cell–cell junction is present, forming slit diaphragms that form a size-selective barrier for the excretion of proteins that is critical for proper kidney filtration [1].

Diabetes mellitus (DM) affected 8.5% of adults worldwide in 2014 [2]. The prevalence of this chronic disorder is increasing. Type 2 diabetes is more common than the autoimmune type 1 form of the disease and consists of 90–95% of DM cases [3]. It is characterized by high blood glucose levels (hyperglycemia), aberrant insulin production, and a decrease in the sensitivity of cells to this hormone [4,5]. Up to 40% of patients with DM develop kidney dysfunction, such as diabetic nephropathy (DN). During the course of DN, many metabolic and histological changes occur. Hyperglycemia results in the generation of reactive oxygen species and oxidative stress that damage DNA, proteins, and lipids in tissues that are affected by the disease [6]. Changes in glycemia and the rate of glycolysis can also lead to a reduction in the formation of foot processes by podocytes, a decrease in their migratory ability, and the induction of apoptosis. This partly results from the fact that glycolysis is the source of adenosine triphosphate (ATP) production in foot processes of podocytes [7] and that hyperglycemic conditions decrease the dependence of podocytes on oxidative phosphorylation and increase the dependence of podocytes on glycolysis [8]. During the course of DN, podocytes start to detach from the GBM [9]. The number of podocytes and slit diaphragms decrease, together with an increase in glycemia and proteinuria, a hallmark of kidney dysfunction [10,11]. Hyperglycemia also leads to the inflammation of renal tissue and its fibrosis. These pathological changes that occur in DN resemble premature cellular senescence [11,12].

Klotho is an anti-aging molecule. Upon its discovery, Klotho was shown to extend its lifespan in mice by 30% and protect them from many disorders, especially disorders of renal tissue [13,14]. Klotho is a transmembrane molecule that can be cleaved to its soluble form by a disintegrin and metalloproteinase domain-containing protein 10 (ADAM-10) as well as ADAM-17, β-secretase 1, and other unknown proteases [13]. Klotho is expressed mostly in the kidneys and brain [13]. Klotho expression was detected in mouse podocytes, cells of the proximal and distal tubules of the nephron, and human glomeruli [15,16]. Moreover, kidney cells were found to be the main source of the soluble form of Klotho, which is considered to be a hormone that exerts beneficial effects on target tissues [17]. This form of the protein was detected in both serum and urine, but reports of Klotho levels in these bodily fluids in renal disorders are rare and sometimes conflicting [13,14,18]. 

Klotho has frequently been reported to decrease inflammation through suppression of the activation of numerous proinflammatory cytokines, chemoattractants, and receptors. Moreover, Klotho inhibits tissue fibrosis [13,19]. Therefore, it can mitigate some pathological changes that occur in diabetes that are caused by hyperglycemia. In mice with streptozotocin-induced type 1 diabetes, Klotho treatment suppressed cardiac inflammation, lowered oxidative stress, and prevented cardiac cell apoptosis [20]. However, only a few studies have investigated its effects on renal tissue under hyperglycemic conditions. Klotho decreases hyperglycemia-induced oxidative stress, with resulting podocyte injury and apoptosis, through the inhibition of various signaling pathways, including insulin-like growth factor 1, protein kinase-1/2, and p38 mitogen-activated protein kinase [21]. Klotho may also prevent protein kinase C-mediated podocyte injury in DN and alleviate glomerular hypertrophy [21,22].

Both membrane-bound and soluble Klotho can bind to fibroblast growth factor (FGF) receptors 1-4 (FGFR1-4), serving as a coreceptor protein for FGF23. Klotho alters the structure of these receptors and increases their binding affinity for FGF23 twenty-fold. FGFRs are widespread throughout the body [23]. Studies in rats and mice have shown that FGFRs are expressed in renal tissue, and FGFR1-3 are expressed in glomeruli [24,25]. However, to our knowledge, no studies have investigated the expression of FGFRs in human podocytes. The function of FGFRs influences the control of phosphate and vitamin D metabolism [14,17,23], the regulation of immune system responses, tissue repair, and tissue regeneration [23,26]. Fibroblast growth factor signaling is also important for cytoskeletal reorganization in podocytes and the formation of their actin-based processes [27]. It is also suggested to be required for podocyte recovery after glomerular injury [28].

The present study analyzed levels of Klotho expression in renal tissue and its levels in serum and urine under both standard and hyperglycemic conditions. Additionally, we investigated whether Klotho influences FGFR1-4 expression and glucose metabolism in podocyte cells that are cultured in media with standard glucose (SG) or high glucose (HG) concentrations. Finally, we investigated whether Klotho protein influences albumin permeability of the GFB. In brief, we found that the decrease in the urinary excretion of Klotho might be an early biomarker of DN and that Klotho administration may have several beneficial effects on renal function in DN.

## 2. Results

### 2.1. Klotho Levels Increase in Serum and Decrease in Renal Tissue and Urine in Diabetic Rats

**Figure 1:**
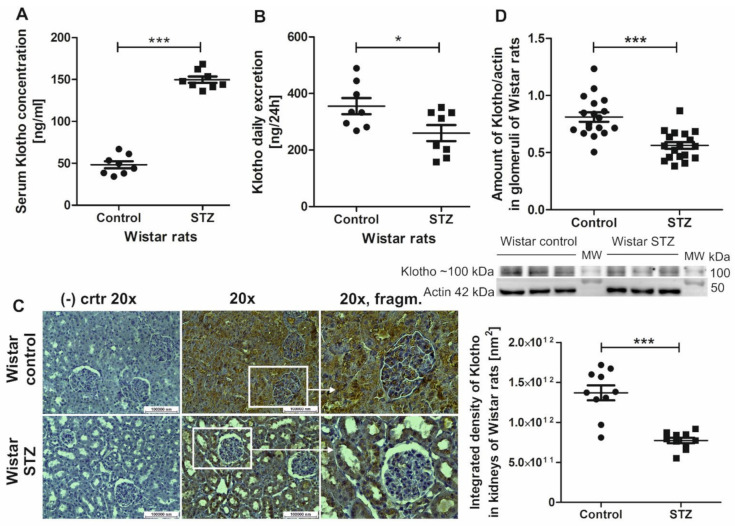
Klotho protein levels increase in serum in Wistar rats with streptozotocin-induced diabetes (STZ) and decrease in their whole kidney tissue, glomeruli, and urine. (**A**) Despite the elevation of Klotho protein levels in serum in STZ rats (*** *p* < 0.0001, vs. control, unpaired *t*-test, *n* = 8), (**B**) a significant decrease in 24-h urinary Klotho excretion was found in STZ rats (* *p* = 0.03, vs. control, unpaired *t*-test, *n* = 8). This may have resulted from (**C**) a significant decrease in Klotho protein levels (renal tissue staining in brown) in glomeruli and cells that form tubules of nephrons in the kidneys in STZ rats (*** *p* < 0.0001, vs. control, unpaired *t*-test, *n* = 10). This was also concordant with (**D**) the downregulation of Klotho protein expression in glomeruli in STZ rats (*** *p* < 0.0001, vs. control, unpaired *t*-test, *n* = 18). (−) ctrl, negative control; MW, molecular weight.

During acute kidney injury, both plasma and urine levels of soluble Klotho protein decrease in affected individuals, which was proposed to be a biomarker of the disease [14]. Therefore, we analyzed the amount of Klotho protein in serum, urine, and renal tissue in Wistar rats with streptozotocin-induced diabetes and age-matched healthy Wistar controls (see Table 1 for a description of the animals). Serum Klotho levels increased by 210% in diabetic rats vs. healthy control rats (149.8 ± 3.89 ng/mL vs. 48.27 ± 4.15 ng/mL, respectively; Figure 1A). Dailey Klotho urine excretion was 27% lower in diabetic rats compared with control rats (259.7 ± 28.34 ng/24 h vs. 355.3 ± 28.45 ng/24 h, respectively; Figure 1B). This could have resulted from a significantly lower (by ~44%) amount of Klotho in renal tissue in STZ rats (integrated density of Klotho labeling in STZ rats vs. control rats: 7.74 × 10^11^ ± 3.47 × 10^10^ nm^2^ vs. 1.37 × 10^12^ ± 9.36 × 10^10^ nm^2^, respectively; Figure 1C). This is consistent with lower (by ~31%) Klotho protein expression in glomeruli from STZ rats compared with control rats (Klotho protein expression normalized to actin: 0.56 ± 0.03 vs. 0.81 ± 0.04, respectively; Figure 1D).

### 2.2. Shedding of Soluble Klotho Increases in Hyperglycemia and Causes a Decrease in the Amount of Podocyte Membrane-Bound Klotho

**Figure 2:**
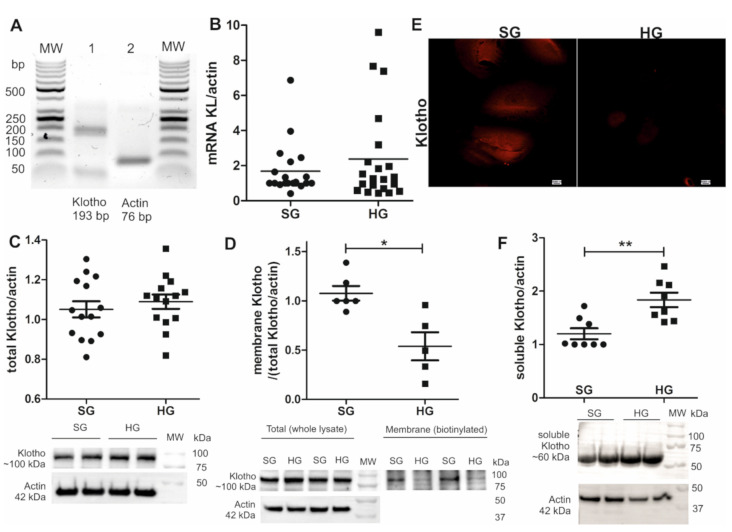
mRNA and total protein levels of Klotho were not downregulated in immortalized human podocytes that were cultured under high glucose (HG) conditions, but the levels of the membrane form of Klotho protein decreased under HG conditions, together with an increase in soluble Klotho shedding. (**A**) The mRNA expression of Klotho was detected in immortalized human podocytes. (**B**) The mRNA expression of Klotho was measured under standard glucose (SG; 11 mM) and high glucose (HG; 30 mM, 5 days) conditions, which did not significantly differ (*p* = 0.96, Mann–Whitney test, *n* = 20–21). (**C**) The total protein expression of Klotho did not differ between podocytes that were cultured under standard glucose and high glucose conditions (*p* = 0.48, unpaired *t*-test, *n* = 14). (**D**) A significant decrease in the levels of the membrane form of Klotho protein was detected in podocytes under HG conditions (* *p* = 0.013, vs. SG, Mann–Whitney test, *n* = 5–6). (**E**) The immunofluorescent labeling of human podocytes for Klotho (red) confirmed the decrease in membrane-bound Klotho protein levels under HG conditions. These results were explained by (**F**) a significant increase in the levels of the soluble form of Klotho under HG conditions (** *p* = 0.007, vs. SG, Mann–Whitney test, *n* = 8), that was cut off from the cell membrane and shed extracellularly. bp, base pairs; MW, molecular weight.

The amount of soluble Klotho protein decreased in urine, and membrane-bound Klotho decreased in renal tissue and glomeruli in diabetic rats (Figure 1). We next analyzed the mRNA and protein expression of Klotho in podocytes, which are a crucial component of the GFB and proper kidney function. Klotho mRNA was expressed in podocytes (Figure 2A), but these levels did not depend on glucose concentration (Figure 2B). The absolute amount of Klotho protein in human podocytes also did not differ under SG and HG conditions (Figure 2C). The level of the podocyte membrane-bound form of Klotho protein decreased by 50% in HG medium (normalized to actin, HG vs. SG: 0.54 ± 0.14 vs. 1.08 ± 0.07; Figure 2D,E), together with a ~53% increase in soluble Klotho shedding (normalized to actin, HG vs. SG: 1.84 ± 0.14 vs. 1.20 ± 0.10; Figure 2F).

### 2.3. FGFRs Are Present on Podocytes and Cells That Form Tubules of the Nephron, and Their Expression Decreases in Hyperglycemia

**Figure 3:**
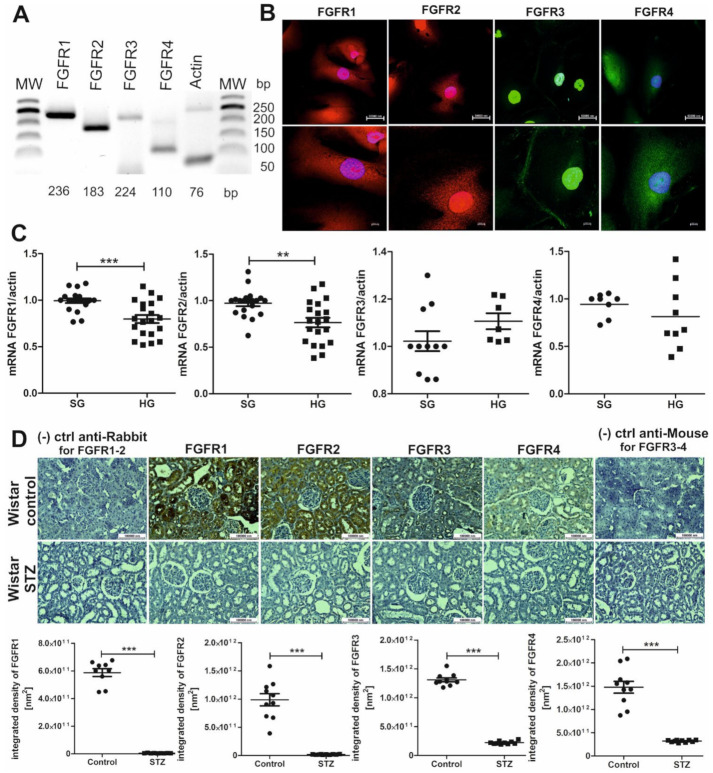
Expression of FGFRs decreases under diabetic conditions in rat kidney tissue and human podocytes. (**A**) *FGFR1*, *FGFR2*, *FGFR3*, and *FGFR4* gene expression was detected in immortalized human podocytes. (**B**) The immunofluorescent labeling of human podocytes for FGFR1-4 revealed their presence in the nucleus, the cytoplasm, and the cell membrane (FGFR1-2 in red; FGFR3-4 in green; DAPI-blue). (**C**) The mRNA expression of *FGFR1* and *FGFR2* genes in human podocytes significantly decreased under high glucose conditions (HG; 30 mM, 5 days) compared with standard glucose conditions (SG; 11 mM; *FGFR1*: *** *p* = 0.0004, unpaired *t*-test, *n* = 19–20; *FGFR2*: ** *p* = 0.0014, unpaired *t*-test, *n* = 20). No significant difference in mRNA expression of the *FGFR3* and *FGFR4* genes was found in human podocytes between SG and HG conditions. (**D**) A decrease in the levels of FGFR1, FGFR2, FGFR3, and FGFR4 was found in renal tissue (stained in brown) in STZ rats compared with healthy control rats (FGFR1-4: *** *p* < 0.0001, unpaired *t*-test, *n* = 9–10). bp, base pairs; (−) ctrl, negative control; MW, molecular weight.

**Figure 4:**
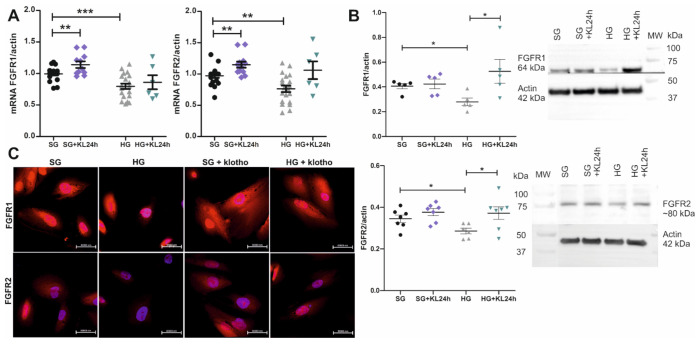
Klotho increases the mRNA and protein expression of FGFRs under standard glucose conditions and recovers it after its decrease under high glucose concentration. (**A**) mRNA expression of the *FGFR1* and *FGFR2* genes increased in human podocytes after 24-h incubation with Klotho (0.5 nM; +KL24h) in the cell medium under standard glucose (SG; 11 mM; *FGFR1*: ** *p* = 0.0075, vs. SG, unpaired *t*-test, *n* = 11–19; *FGFR2*: ** *p* = 0.0038, vs. SG, unpaired *t*-test, *n* = 12–20). A trend was also found toward the rescue of *FGFR1* and *FGFR2* gene expression by 24-h incubation with Klotho after its initial drop under high glucose conditions (HG, 30 mM glucose, 5 days; HG+KL24h vs. HG). Detailed statistical data for the SG vs. HG comparisons are presented in Figure 3 (*FGFR1*: *** *p* = 0.0004; *FGFR2*: ** *p* = 0.0014). (**B**) Protein levels of FGFR1 and FGFR2 increased in human podocytes that were incubated with Klotho for 24-h (normalized to actin, HG+KL24h vs. HG: FGFR1: * *p* = 0.04, unpaired *t*-test, *n* = 5; FGFR2: * *p* = 0.03, unpaired *t*-test, *n* = 6–7; Figure 4B) after its initial drop under hyperglycemic conditions (normalized to actin, HG vs. SG: FGFR1: * *p* = 0.01, unpaired *t*-test, *n* = 5; FGFR2: * *p* = 0.03, unpaired *t*-test, *n* = 6–7; Figure 4B). (**C**) An increase in the protein expression of FGFR1 and FGFR2 after 24-h incubation with Klotho under both SG and HG conditions was also observed by the immunofluorescent staining of immortalized human podocytes (FGFR1-2 in red; DAPI-blue). MW, molecular weight.

The level of membrane-bound Klotho in podocytes decreases under hyperglycemic conditions. Klotho interacts with FGFR1-4. We analyzed whether they are also present on human podocytes, whether their level changes under diabetic conditions (Figure 3), and whether Klotho impacts the level of their expression (Figure 4). The mRNA expression of genes that encode FGFR1, FGFR2, FGFR3, and FGFR4 was detected in human podocytes (Figure 3A). The immunofluorescent labeling of these receptor proteins in human podocytes showed that they were localized throughout the whole cell (i.e., in the nucleus, cytoplasm, and cell membrane; Figure 3B). Under diabetic conditions, mRNA expression of the *FGFR1* and *FGFR2* genes significantly decreased by 20% and 21.5%, respectively (expression normalized to actin, HG vs. SG: *FGFR1*: 0.80 ± 0.04 vs. 0.99 ± 0.02, respectively; *FGFR2*: 0.76 ± 0.05 vs. 0.97 ± 0.03, respectively; Figure 3C). The decrease in FGFR1 and FGFR2 expression in podocytes that was caused by hyperglycemia was also confirmed at the protein level, which decreased by 32% and 15%, respectively, compared with SG conditions (normalized to actin, HG vs. SG: FGFR1: 0.28 ± 0.03 vs. 0.41 ± 0.02, respectively; FGFR2: 0.29 ± 0.01 vs. 0.34 ± 0.02, respectively; Figure 4B). Furthermore, a decrease in the protein expression of all four FGFRs was found in renal tissue in diabetic rats vs. controls, with a >99% decrease in FGFR1 (5.18 × 10^9^ ± 4.01 × 10^8^ nm^2^ vs. 5.81 × 10^11^ ± 2.84 × 10^10^ nm^2^), >98% decrease in FGFR2 (1.85 × 10^10^ ± 1.56 × 10^9^ nm^2^ vs. 9.87 × 10^11^ ± 1.09 × 10^11^ nm^2^), ~83% decrease in FGFR3 (2.23 × 10^11^ ± 8.44 × 10^9^ nm^2^ vs. 1.31 × 10^12^ ± 3.34 × 10^10^ nm^2^), and >78% decrease in FGFR4 (3.21 × 10^11^ ± 7.38 × 10^9^ nm^2^ vs. 1.48 × 10^12^ ± 1.27 × 10^11^ nm^2^; Figure 3D).

### 2.4. The Addition of Klotho Increases FGFR1 and FGFR2 Expression

mRNA expression of the *FGFR1* and *FGFR2* genes significantly decreased under hyperglycemic conditions. We next investigated whether Klotho upregulates the expression of FGFR1 and FGFR2 in human podocytes. The addition of Klotho for 24 h to the cell culture medium (+KL24h) caused a ~15% and ~18% increase in the mRNA expression of *FGFR1* and *FGFR2*, respectively, in human podocytes that were grown under SG conditions (normalized to actin, SG+KL24h vs. SG: *FGFR1*: 1.14 ± 0.05 vs. 0.99 ± 0.02, respectively; *FGFR2*: 1.15 ± 0.05 vs. 0.97 ± 0.03, respectively; Figure 4A). The addition of Klotho to the HG medium increased the mRNA expression of *FGFR2* by 39% in podocytes, but this effect did not reach statistical significance (normalized to actin, HG+KL24h vs. HG: 1.06 ± 0.14 vs. 0.76 ± 0.05, respectively; *p* = 0.07, Mann–Whitney test, *n* = 6–20; Figure 4A). This tendency was also observed for the *FGFR1* gene, but it was less prominent than for *FGFR2*. The increase in *FGFR1* and *FGFR2* gene expression in human podocytes after the addition of Klotho to the HG medium for 24 h translated into significant 89% and 28% increases in the FGFR1 and FGFR2 protein levels, respectively (normalized to actin, HG+KL24h vs. HG: FGFR1: 0.53 ± 0.10 vs. 0.28 ± 0.03, respectively; FGFR2: 0.37 ± 0.03 vs. 0.29 ± 0.01, respectively; Figure 4B). These results were confirmed by the immunofluorescent labeling of human podocytes, with a visible positive effect of the addition of Klotho on the increase in FGFR1 and FGFR2 protein levels in cells that were grown under both SG and HG conditions (Figure 4C).

### 2.5. Klotho Increases Glycolysis and Glycolytic Capacity Levels in Podocytes That Are Grown under Both SG and HG Conditions

**Figure 5:**
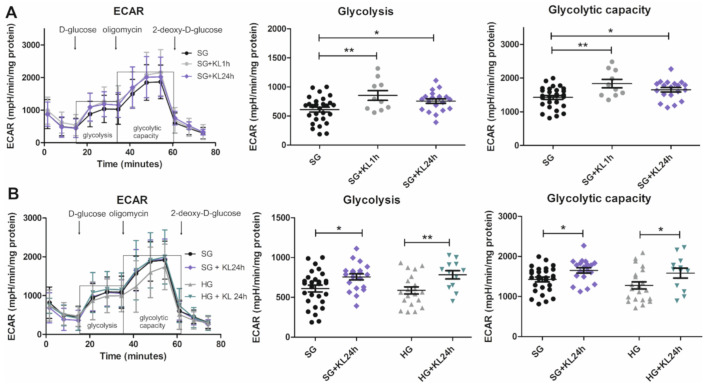
Klotho increases glycolysis and glycolytic capacity in immortalized human podocytes. (**A**) The addition of Klotho (0.5 nM) for 1 h (+KL1h) and 24 h (+KL24h) to the cell medium with standard glucose (SG; 11 mM) significantly increased glycolysis (SG+KL1h vs. SG: ** *p* = 0.008, unpaired *t*-test, *n* = 10–28; SG+KL24h vs. SG: * *p* = 0.018, unpaired *t*-test, *n* = 20–28) and glycolytic capacity (SG+KL1h vs. SG: ** *p* = 0.002, unpaired *t*-test, *n* = 10–28; SG+KL24h vs. SG: * *p* = 0.017, unpaired *t*-test, *n* = 19–28) in human podocytes. (**B**) The addition of Klotho for 24 h (+KL24h) to the cell medium with both standard and high glucose (HG; 30 mM, 5 days) increased glycolysis (SG+KL24h vs. SG: ut supra; HG+KL24h vs. HG: ** *p* = 0.009, unpaired *t*-test, *n* = 12–20) and glycolytic capacity (SG+KL24h vs. SG: ut supra; HG+KL24h vs. HG: * *p* = 0.05, unpaired *t*-test, *n* = 12–21) in human podocytes.

As mentioned above, disruptions of glycolysis can be caused by hyperglycemia and can affect the function of the GFB [7,8]. Based on the changes in Klotho protein levels under hyperglycemic conditions, we next investigated whether it impacts glycolytic parameters in podocytes. The addition of recombinant Klotho protein to the SG culture medium increased the level of glycolysis and glycolytic capacity in podocytes after both 1 h (+KL1h) and 1 day (+KL24h) of incubation (glycolysis: ~40% increase in SG+KL1h vs. SG, 853.80 ± 84.45 mpH/min/mg protein vs. 610.60 ± 42.24 mpH/min/mg protein, respectively; ~24% increase in SG+KL24h vs. SG, 757.20 ± 38.40 mpH/min/mg protein vs. 610.60 ± 42.24 mpH/min/mg protein, respectively; glycolytic capacity: ~29% increase in SG+KL1h vs. SG, 1836 ± 124.50 mpH/min/mg protein vs. 1426 ± 60.06 mpH/min/mg protein, respectively; ~16% increase in SG+KL24h vs. SG, 1652 ± 66.25 mpH/min/mg protein vs. 1426 ± 60.06 mpH/min/mg protein, respectively; Figure 5A). Incubation with Klotho for 24 h increased glycolysis and glycolytic capacity under both SG and HG conditions (glycolysis: ~33% increase in HG+KL24h vs. HG, 783.80 ± 52.30 mpH/min/mg protein vs. 587.9 ± 44.44 mpH/min/mg protein; glycolytic capacity: ~24% increase in HG+KL24h vs. HG, 1585 ± 124.40 mpH/min/mg protein vs. 1277 ± 89.42 mpH/min/mg protein; Figure 5B).

### 2.6. Klotho Improves Function of the GFB

**Figure 6:**
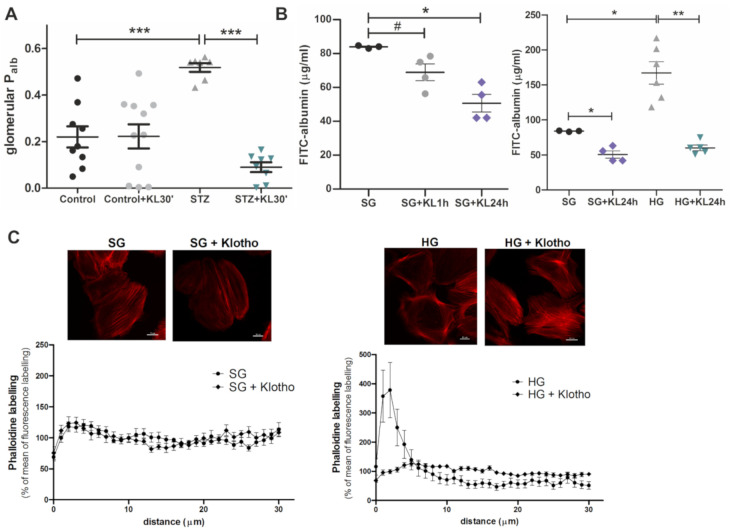
Klotho exerted a beneficial effect on the maintenance of proper permeability of the glomerular filtration barrier. (**A**) The albumin permeability of glomeruli from Wistar rats with streptozotocin-induced diabetes (STZ) significantly increased compared with glomeruli from healthy Wistar control rats (*** *p* = 0.0007, Mann–Whitney test, *n* = 7–9). Thirty minute incubation with Klotho (0.5 nM; +KL30′) significantly decreased the albumin permeability of glomeruli from STZ rats (*** *p* = 0.0003, Mann–Whitney test, *n* = 7–8). (**B**) Klotho decreased the albumin permeability of a human podocyte monolayer under standard glucose conditions (SG; 11 mM) after 24-h incubation (+KL24h; * *p* = 0.049, vs. SG, Mann–Whitney test, *n* = 3–4), with a less prominent effect after 1-h incubation (+KL1h); # *p* ~ 0.05 but above it. Twenty-four hour incubation with Klotho had a significant impact on lowering albumin permeability of the human podocyte monolayer, which significantly increased under high glucose conditions (HG; 30 mM, 5 days) compared with standard glucose conditions (HG vs. SG: * *p* = 0.024, Mann–Whitney test, *n* = 3–6; HG+KL24h vs. HG: ** *p* = 0.004, Mann–Whitney test, *n* = 5–6). (**C**) Klotho (0.5 nM, 24 h) reversed HG-induced changes in cytoskeletal (F-actin) reorganization in immortalized human podocytes. Scale bar = 20 μm. Digitized fluorescence images of phalloidin-stained (far red) podocytes were used to obtain the fluorescence intensity profiles of the F-actin network. The data are expressed as mean ± SEM (*n* = 9–16).

During the pathological DN process, permeability of the GFB increases to the point that large proteins, such as albumin, can pass through the barrier and be excreted in urine [11]. The increase in albumin permeability is a consequence and hallmark of renal dysfunction. As mentioned above, Klotho increases glycolysis and glycolytic capacity in human podocytes, disruptions of which can alter GFB function [7]. We next investigated whether Klotho affects albumin permeability in rat glomeruli and human podocytes under hyperglycemic and control conditions. Albumin permeability in glomeruli from diabetic rats was 136% higher than in control rats (0.52 ± 0.02 vs. 0.22 ± 0.06, respectively; Figure 6A). The incubation of glomeruli from diabetic rats with Klotho (0.5 nM) for 30 min (+KL30′) decreased albumin permeability by 83% compared with glomeruli that were not treated with Klotho (0.09 ± 0.02 vs. 0.52 ± 0.02, respectively; Figure 6A). This effect of Klotho was also found for a monolayer of human podocytes, the albumin permeability of which increased by 99% under hyperglycemic conditions (HG vs. SG: 167.10 ± 15.93 vs. 83.98 ± 0.48, respectively). The incubation of podocytes with Klotho (0.5 nM) for 24 h (+KL24h) decreased albumin permeability under both SG (by 40%) and HG (by 64%) conditions (SG+KL24h vs. SG: 50.65 ± 5.23 vs. 83.98 ± 0.48, respectively; HG+KL24h vs. HG: 60.17 ± 4.07 vs. 167.10 ± 15.93, respectively; Figure 6B). The attenuation in HG-induced albuminuria by Klotho may be explained by the effect of Klotho on F-actin cytoskeleton reorganization in human podocytes. As revealed by the phalloidin staining of F-actin distribution in podocytes, HG caused F-actin redistribution to cortical regions of the cell, whereas the addition of Klotho (0.5 nM) for 24 h restored F-actin distribution throughout the podocyte (Figure 6C).

## 3. Discussion

In the present study, we found a significant decrease in the membrane expression of Klotho in glomeruli and renal tissue in diabetic rats. These findings are consistent with the fact that, in patients with chronic kidney disease (CKD), low levels of calcitriol are found, which is an activator of Klotho expression [29]. We found that the decrease in renal Klotho expression was accompanied by the lower urine excretion of soluble Klotho in early diabetic rats, which accumulated inside the body and increased in serum. Conflicting findings of plasma and urine levels of Klotho have been reported in individuals with DN. Such disparate findings have been reported by studies that included various groups with different stages of DN [18,30,31]. Kacso et al., also reported a decrease in urinary levels of Klotho protein in a group of patients with DN. However, similar to the study of Bob et al., the group of patients had different stages of nephropathy [30]. Bob et al., reported that the increase in serum levels of soluble Klotho was linked to a rapid annual decline of kidney function [31]. According to van Ark et al., circulating levels of Klotho protein were not disrupted in serum from patients with type 2 diabetes without nephropathy, suggesting that circulating Klotho protein levels might be a hallmark of DN [32]. Cho et al., reported that individuals with low urinary Klotho levels, similar to the diabetic rats in the present study, were significantly more prone to have foot process effacement, whereas high serum Klotho levels were associated with a lower risk of interstitial fibrosis and segmental glomerulosclerosis [33]. This would reflect pathological changes that are observed during initial stages of DN that may be biomarkers of the disease, which is consistent with our aforementioned findings in diabetic rats 14 days after diabetes induction by STZ.

Kim et al., reported the presence of Klotho in human glomeruli [15]. To our knowledge, the present study is the first to report the presence of Klotho in human podocytes. The mRNA and total protein expression of Klotho were unaltered under hyperglycemic conditions. However, we found that the level of the membrane form of Klotho significantly decreased under hyperglycemic conditions, together with an increase in soluble Klotho shedding. This could be explained by the increased level of serum ADAM10 and the elevated kidney expression of ADAM17 in diabetic models, which are both Klotho-shedding metalloproteinases [34,35]. This was also consistent with our finding of the increase in soluble Klotho levels in serum in diabetic rats and the previous finding that the majority of Klotho shedding occurs in the kidneys [17]. The lower level of membrane-bound Klotho under hyperglycemic conditions was previously reported for distal convoluted tubules of the nephron but not for podocytes [36]. This may be correlated with the previously reported aggravation of diabetes-induced oxidative stress, inflammation, podocyte injury, and apoptosis, resulting in proteinuria, that are caused by Klotho deficiency [13,22].

We also found that all four FGFRs were present in human podocytes at both the mRNA and protein levels. Under hyperglycemic conditions, we observed a significant decrease in the protein levels of FGFR1-4 and the mRNA expression of *FGFR1* and *FGFR2*. We also found that the addition of recombinant Klotho to the cell medium increased the mRNA expression of *FGFR1* and *FGFR2* under SG conditions, with an upward tendency under hyperglycemic conditions. This tendency accelerated at the protein level, in which the addition of Klotho significantly increased the expression of both FGFR1 and FGFR2 in podocytes that were grown in HG, which was also confirmed by immunofluorescent staining.

Studies of the level and function of FGFRs in renal tissue in diabetic individuals are scarce. Consistent with the present findings, Taylor et al., reported a decrease in the protein level of FGFR4 in mice with CKD [37]. Cheng et al., reported a decrease in the expression of FGFRs in the kidneys in diabetic rats, which can be due to an increase in the presence of ADAM10/17 metalloproteinases, which have been recently proven to cleave FGFRs [38,39]. Chen et al., however, found no effect of Klotho on FGFR expression, like we did [38]. Klotho upregulated FGFR levels in the present study, which may reflect a beneficial effect against DN. According to Wu et al., the activation of FGFR1 by an antibody that mimicked FGF21 ameliorated hyperglycemia in type 2 diabetes [40]. FGFR2 was found to be important in protection against the apoptosis of tubular cells in acute kidney injury, partly by stimulating the activation of extracellular signal-regulated kinase 1/2 (Erk1/2; also called mitogen-activated protein kinase 3/1) [41]. Cheng et al., suggested that the restoration of FGFR levels in the kidneys in diabetic rats can protect against fibrosis [38].

In the present study, Klotho increased glycolysis and glycolytic capacity in human podocytes under both standard and hyperglycemic conditions. To our knowledge, the only studies that investigated the influence of Klotho protein at the level of glycolysis were performed under aerobic conditions and with organs other than the kidneys. In the brain, full-length Klotho protein participates in supplying nutrients to neurons by astrocytes. Klotho stimulates a rapid increase in aerobic glycolysis and lactate release from astrocytes, which is needed for energy production by neurons [42]. However, as reported by Bringkoetter et al., podocytes primarily rely on anaerobic glycolysis and, to a lesser extent, on the β-oxidation of lipids [43]. In the present study, both aerobic and anaerobic glycolysis were increased by Klotho in podocytes, in which the upregulation of glycolytic capacity by Klotho mirrored anaerobic glycolysis conditions and oxidative phosphorylation was halted by an injection of oligomycin [42,44,45]. As mentioned above, ATP deficiency in podocytes results in a decrease in the formation of foot processes, a decrease in the migratory ability of the cells, and the induction of apoptosis [7], which can lead to defective glomerular filtration and nephropathy. Therefore, the increase in glycolytic parameters that is induced by Klotho might enable podocytes to withstand the damaging effects of hyperglycemia. As revealed in adipocytes, glycolysis produces metabolites for lipogenesis and directs fatty acids from excessive oxidation to the synthesis of triglycerides, thereby reducing oxidative stress. Such a beneficial function of glycolysis is consistent with the function of Klotho protein [13,46].

Our research group previously found that HG conditions increase albumin permeability in rat glomeruli and cultured rat podocytes, accompanied by F-actin redistribution to cortical regions of podocytes [47,48,49]. However, to our knowledge, the present study is the first report of an analogous effect of hyperglycemia on human podocytes. We also found that the incubation of rat glomeruli and a monolayer of human podocytes with recombinant Klotho significantly decreased albumin permeability, especially under hyperglycemic conditions. This may be linked to our observation that Klotho reversed the HG-induced redistribution of F-actin filaments, the organization of which is critical for proper glomerular filtration [15]. Consistent with our findings, Klotho ameliorated ATP-induced reorganization of the actin cytoskeleton in mouse podocytes and decreased proteinuria by targeting transient receptor potential channel 6 (TRPC6) [15]. Moreover, a previous study reported that Klotho caused functional and histological improvements of renal tissue [50]. Thus, the addition of Klotho may prevent proteinuria and restore function of the GFB in DN.

To strengthen the findings of the current study, future analyses should concern testing of human serum and/or urine samples for Klotho level assessment. These analyses could also show results of iv vivo-based studies of FGF receptors levels, glycolytic parameters and albumin permeability of glomeruli and podocytes after Klotho supplementation in diabetic rats.

## 4. Materials and Methods

### 4.1. Ethical Approval

The experiments were performed with male Wistar rats (167–342 g) that were obtained from the Mossakowski Medical Research Centre, Polish Academy of Sciences, Warsaw, Poland. The rats were maintained on a 12 h/12 h light/dark cycle with free access to a standard pellet diet and tap water. The experiments were conducted in accordance with directive 2010/63/EU, and the protocol was approved by the local Ethical Commission at the University of Science and Technology in Bydgoszcz, Poland (no. 54/2018).

### 4.2. Experimental Animals and Metabolic Cage Studies

The experiments were conducted with male rats that were subjected to diabetes and induced by streptozotocin (80 mg/kg, i.p.), and age-matched control Wistar rats. In diabetic rats, the experiments were performed 14 days after diabetes induction, only in animals with fasting blood glucose concentrations >250 mg/dL (Table 1). Blood samples (for serum Klotho analysis) were taken from the animals shortly after. Subsequently, the rats were kept in separate metabolic cages for 48 h (15th to 17th day after diabetes induction) with free access to a regular pellet diet and drinking water. The animals were first allowed to habituate to the cages for 24 h. During the next 24 h, urine was collected, and the urinary excretion of albumin and soluble Klotho was measured in an external laboratory and using an enzyme-linked immunosorbent assay (ELISA; described below), respectively. Fasting blood glucose levels were measured in whole samples using a glucose oxidase meter (Accu-Chek Performa, Roche Diagnostics, Mannheim, Germany). In the 17th day after diabetes induction, rats were sacrificed to isolate glomeruli from their kidneys.

### 4.3. Isolation of Rat Glomeruli

Male Wistar rat kidneys were removed and placed in ice-cold phosphate-buffered saline (PBS; pH 7.4) supplemented with 0.49 mM MgCl_2_, 0.9 mM CaCl_2_, and 5.6 mM glucose. The renal capsule was removed, and a cortex was minced with a razor blade and then pressed through a series of sieves with decreasing pore diameters (250, 125, and 75 μm). The obtained cell suspension contained decapsulated glomeruli without afferent and efferent arterioles. The entire procedure was performed in an ice bath and completed in less than 1 h.

### 4.4. Human Podocyte Cell Culture

Human immortalized podocytes (kind gift from Moin A. Saleem) were generated, as described previously [51]. The expression of podocyte markers and cell line purity were checked regularly [48]. Podocytes were grown in RPMI1640 medium (Thermo Fisher Scientific, Waltham, MA, USA) supplemented with heat-inactivated fetal bovine serum (Thermo Fisher Scientific) and antibiotics (penicillin/streptomycin, Sigma Aldrich, St. Louis, MO, USA). Cells were grown at 33 °C for proliferation to the desired confluence for 10–15 days, at 37 °C for cell differentiation, and then under the respective experimental conditions for the next 5 days. During the last 5 days of culture, the cells were grown with standard glucose (SG; 11 mM) or HG (30 mM for 5 days) alone in the medium or with SG or HG with the addition of the active, soluble form of Klotho protein (Abcam, Cambridge, UK, catalog no. AB84072) to a final concentration of 0.5 nM for 1 h or 24 h.

### 4.5. Biotinylation

To assess the amount of membrane Klotho protein in human podocytes, cells were incubated for 30 min with biotin solution (Thermo Fisher Scientific, catalog no. 21338) in PBS (pH 8.0). Excess biotin was washed out with PBS with the addition of glycine (100 mM, pH 8.0). Subsequently, the cells were lysed, as described previously [52]. A portion of the lysate was frozen for further assessments of total Klotho protein levels in podocytes. The majority of the lysate was mixed with Neutr/Avidin resin (Thermo Fisher Scientific, catalog no. 53150) and incubated on a rotor at 4 °C overnight. The next day, Neutr/Avidin resin with bound biotinylated membrane proteins was washed five times with PBS and heated at 96 °C for 10 min with 2× concentrated loading buffer (0.5M Tris, 10% sodium dodecyl sulfate (SDS), 30% glycerol, 9.3% DL-dithiothreitol, and 0.012% bromophenol blue). The samples were then analyzed by Western blot together with the portion of the lysate that contained total Klotho in podocytes (heated at 96 °C for 2 min in 1× concentrated loading buffer).

### 4.6. Western Blot

Human podocytes and rat glomerulus lysates were prepared, as described previously [49,52]. Amicon Ultra Centrifugal Filters (catalog no. UFC201024, Millipore, Burlington, MA, USA) were used according to the manufacturer’s instructions to concentrate proteins in the cell medium on a layer of human podocytes. After initial heating at 96 °C for 2 min, equal amounts of total protein (20 μg/well for glomerulus lysates and 30 or 40 μg/well for podocyte lysates and concentrate of proteins in the cell medium) underwent SDS-polyacrylamide gel electrophoresis (PAGE) and immunoblotted on polyvinyl difluoride membranes. The primary antibodies that were used are listed in Table 2. The secondary antibodies were anti-rabbit (catalog no. A9169, Sigma Aldrich) and anti-mouse (catalog no. A9044, Sigma Aldrich). Densitometric quantification of the obtained bands was performed using ImageJ 1.52a software (National Institutes of Health, Bethesda, MD, USA).

### 4.7. Immunohistochemistry

Formalin-fixed paraffin-embedded kidney specimens that were obtained from Wistar control and Wistar STZ rats were deparaffined with Histochoice Cleaning Agent (Sigma Aldrich) and ethanol and hydrated in water. Epitopes were retrieved by incubating the specimens in sodium citrate buffer (10 mM, pH 6.0) at 97 °C for 20 min. The specimens were blocked with 5% bovine serum albumin (BSA) for 1 h. Bovine serum albumin was then removed from the slides, and primary antibodies (diluted in 5% BSA) were added to the specimens, followed by overnight incubation at 4 °C (Table 2). The next day, the specimens were incubated with secondary antibodies (Cell Signaling Technology, Danvers, MA, USA, catalog no. 8114S or 8125S) at room temperature for 30 min. The slides were then incubated for 2 min with Signal Stain DAB substrate (Cell Signaling Technology). The specimens were counterstained with hematoxylin for 5 s and dehydrated with ethanol and Histochoice. Cells were imaged using a Nikon Eclipse Ti microscope. Quantification of the amount of stained proteins was performed using ImageJ 1.52a software (National Institutes of Health).

### 4.8. ELISA

A commercially available Rat Klotho ELISA Kit (Fine Test, Wuhan Fine Biotech, Wuhan, Hubei, China; catalog no. ER0658) was used to determine concentrations of soluble Klotho in serum and urine samples from Wistar rats. Twenty-four hour urinary Klotho excretion was calculated based on urine Klotho concentration and the volume of 24-h urine excretion. The assay was performed in accordance with the manufacturer’s instructions. Data was analyzed using MyAssay software (https://myassays.com/) (accessed on 3 August 2020).

### 4.9. Real-Time Polymerase Chain Reaction

Total cellular RNA was isolated from human podocytes using the RNeasy Plus Mini Kit, including an initial step of genomic DNA elimination (Qiagen, Hilden, Germany). Quantity and purity of the obtained RNA were determined using a NanoDrop device (Thermo Fisher Scientific). A reverse transcription reaction was performed on isolated RNA. The obtained complementary DNA was subjected to a real-time polymerase chain reaction (PCR) that was performed using a LightCycler 480 instrument (Roche, Basel, Switzerland) with gene-specific intron-spanning primers and 8 bp fluorescent hydrolysis probes (Roche; Table 3). Relative quantification of the initial amount of specific mRNA transcripts was performed using the ΔΔCt method, with β-actin as a reference. The amplified products were separated on agarose gel (2.5%) and imaged using a Molecular Imager with Image Lab 6.0 software (Bio-Rad, Hercules, CA, USA).

### 4.10. Immunofluorescent Staining

Podocytes were grown on glass coverslips that were coated with type I collagen. After incubation under experimental conditions, the cells were fixed with 4% paraformaldehyde, permeabilized with 0.1% Triton-X 100, and blocked with 3% BSA in PBS (blocking solution). Subsequently, the cells were incubated overnight at 4 °C with primary antibodies (Table 2) that were diluted in 3% BSA solution. The next day, the cells were incubated with secondary antibodies (Thermo Fisher Scientific, catalog no. A11010 or A11059; 1:200 dilution in blocking solution) for 2 h at 4 °C. Afterward, cell nuclei were labeled with 1:1000 DAPI solution (Bio-Rad, catalog no. 135-1303) in PBS in the dark for 15 min at room temperature. The cells were imaged using a re-scan confocal microscope (Eclipse Ti, Nikon Instruments, Melville, NY, USA; RCM device, Confocal.nl, Amsterdam, The Netherlands).

### 4.11. Glycolysis Stress Test

A glycolysis stress test was performed using a Seahorse XFp analyzer (Agilent Technologies, Santa Clara, CA, USA), which depends on measurements of the extracellular acidification rate (ECAR). The ECAR increases through proton release from cells to the assay medium, which occurs during the glycolysis process. Glycolytic parameters that are obtained in the analysis are mainly levels of glycolysis and glycolytic capacity [53]. First, the ECAR was measured for cells that were starved with a low level of glycolysis substrates (1 h in minimal Dulbecco’s modified essential medium supplemented with 2 mM L-glutamine). Second, glucose was injected into the assay medium to a final concentration of 10 mM to analyze glycolysis levels. Third, oligomycin (final concentration: 1 µM) was injected into the medium, which enabled glycolytic capacity measurements in the analyzed cells. The final step of the analysis was an injection of 2-deoxyglucose (final concentration: 50 mM), which results in instant glycolysis inhibition, together with a decrease in the ECAR. Each measurement was taken three times, every three min. The results were corrected for protein concentration (in milligrams) in each well of the assay miniplate using the Bradford method.

### 4.12. Glomerular Permeability to Albumin In Vitro

The volume response of glomerular capillaries to an oncotic gradient that was generated by defined concentrations of albumin was measured, as described previously [54]. Briefly, glomeruli were isolated from male diabetic and age-matched control Wistar rats. Afterward, they were affixed to 0.1% poly-L-lysine-coated plates. Unattached glomeruli were removed by a gentle wash with fresh medium (PBS supplemented with 0.49 mM MgCl_2_, 0.9 mM CaCl_2_, and 5.6 mM glucose) that contained 5% BSA. Adherent glomeruli were then incubated in 5% BSA medium that contained Klotho protein (0.5 nM) for 30 min at 37 °C. The initial incubation medium was replaced with a medium that contained 1% BSA to produce an oncotic gradient across the glomerular capillary wall (5% BSA in the lumen vs. 1% BSA in the bath medium). Control glomeruli were treated with equivalent volumes of medium that contained 5% BSA (no oncotic gradient). Glomerular volume changes were recorded by video microscopy (Olympus IX51, Olympus, Tokyo, Japan) before and after a medium change to an oncopressive medium (1% BSA). Glomerular volume (V) was calculated from the surface area (A) of the glomerulus using the following formula using Olympus CellSens Dimension software: V = (4/3A$\sqrt{A/\pi }$)/10^6^. Volume changes (ΔV) were calculated as ΔV = (V_final_ − V_initial_)/V_initial_ based on the direct relationship between the increase in ΔV and oncotic gradient that was applied across the capillary wall. This principle was used to calculate the reflection coefficient of albumin (σ_alb_), defined as the ratio of ΔV for experimental glomeruli to ΔV of control glomeruli in response to identical oncotic gradients, where σ_alb_ = ΔV_experimental_/ΔV_control_. The reflection coefficient of albumin (convectional P_alb_) was used to calculate the glomerular capillary permeability to albumin (1 − σ_alb_), which describes the movement of albumin consequent to water flow. 

### 4.13. Albumin Permeability of Human Podocytes

The transepithelial albumin permeability of human podocyte cells was assessed by measuring FITC-labeled BSA diffusion through a podocyte monolayer, as previously described [55], with minor changes [52]. Podocytes were grown on permeable supports with a collagen-coated 3.0 µm membrane (Corning, Corning, NY, USA, catalog no. 3496) and placed in 24-well plates. Two hours before the experiment, the cell medium was replaced with serum-free medium (SFM). The SFM in the upper compartment was subsequently replaced with a 0.2 mL portion of fresh SFM. The SFM in the lower compartment was replaced with 1.2 mL of SFM that contained 1 mg/mL FITC-albumin. After 1 h of incubation, a 200 μL sample of the solution from the upper chamber was transferred to a 96-well plate, and the concentration of FITC-albumin was evaluated based on the measurement of its absorbance at 490 nm in a plate spectrophotometer (ELx808, BioTek Instruments, Winooski, VT, USA).

### 4.14. F-Actin Network

To visualize the F-actin network in human podocytes that were cultured on human fibronectin-coated coverslips, Alexa Fluor 633 Phalloidin (Thermo Fisher Scientific, catalog no. A22284) was used. NIS-Elements General Analysis 3 software (Nikon) was used to generate fluorescence intensity profiles from the basal membrane to the nucleus. The normalization of fluorescent profiles of different cells was performed. The fluorescence intensity on the *X*-axis at a 1 μm distance is expressed as a percentage of the mean value of the fluorescence intensity that was calculated for the total *X*-axis. The cell membrane was positioned at point 0 on the plot.

### 4.15. Statistical Analysis

The data were analyzed using Prism 5.03 software (GraphPad, San Diego, CA, USA). Datasets were tested for a Gaussian distribution using the Shapiro–Wilk test. Depending on the results of this test, the statistical significance of the data was analyzed using parametric tests (parametric analysis of variance (ANOVA) followed by Bonferroni’s multiple-comparison *post hoc* test, unpaired *t*-test) or nonparametric tests (the Kruskal–Wallis test followed by Dunn’s multiple-comparison *post hoc* test and the Mann–Whitney test). Values of *p* ≤ 0.05 were considered statistically significant. The results are expressed as mean ± SEM.

## 5. Conclusions

In conclusion, we observed significantly higher serum levels of soluble Klotho and a decrease in its urinary excretion, which may be considered early biomarkers of DN. We found that Klotho improved the function of renal tissue through effects on the restoration of FGFRs, improved glycolysis, and lowered albumin permeability under hyperglycemic conditions. Our findings indicate that Klotho should be investigated further with regard to its potential role in reducing the pathological effects of DN.

## Figures and Tables

**Table 1:** Metabolic balance studies in healthy male Wistar rats (Control) and age-matched Wistar rats with streptozotocin-induced diabetes (STZ).

Parameter	Control *n* = 4	STZ *n* = 4
Body weight (g)	286 ± 23.98	210.25 ± 19.80
Blood glucose (mg/dl)	112.75 ± 4.87	422 ± 65.40 *
Urine volume (ml/24 h)	9.5 ± 1.51	166.5 ± 10.87 *
Urinary albumin excretion (mg/24 h)	0.15 ± 0.03	2.57 ± 0.67 *

The data are expressed as mean ± SEM. Glucose levels were elevated in blood in diabetic STZ Wistar rats, and the volume of their urine and urine albumin excretion were increased, compared with control rats. * *p* = 0.03, for all comparisons.

**Table 2:** Primary antibodies that were used in the experiments.

Antibody	Clonality	Dilution	Source
Klotho	monoclonal	1:50 (IF); 1:400 (IHC); 1:445 (WB)	Sigma Aldrich, SAB3500604
Soluble Klotho	monoclonal	1:500 (WB)	Cusabio Technology, CSB-PA552336
FGFR1	polyclonal	1:50 (IF); 1:30 (IHC); 1:2000 (WB)	Cusabio Technology, CSB-PA008642LA01HU
FGFR2	polyclonal	1:25 (IF); 1:25 (IHC); 1:500 (WB)	Cusabio Technology, CSB-PA000992
FGFR3	monoclonal	1:15 (IF); 1:20 (IHC)	Santa Cruz Biotechnology, sc-390423 (IF), sc-13121 (IHC)
FGFR4	monoclonal	1:15 (IF); 1:15 (IHC)	Santa Cruz Biotechnology, sc-136988
Actin	monoclonal	1:5000 (WB)	Sigma Aldrich, A5441

IF, immunofluorescence; IHC, immunohistochemistry; WB, Western blot.

**Table 3:** Sequences of primers and fluorescent probes that were used in the experiments.

Gene Name	Accession no. for mRNA Sequence	Primer Sequence	Probe Sequence	Product Length
*KL*	NM_004795.4	F: GCTCAACTCCCCCAGTCAGG R: TGTGGGCTTTGAGAGCTTCG	CCAGGGCA	193
*FGFR1*	NM_001174063.2	F: CTTAGGCAAACCCCTGGGAG R: ACAAGGGACCATCCTGCG	CTGCTGGG	236
*FGFR2*	NM_000141.5	F: CCGTGAAGATGTTGAAAGATGATGC R: GGTATTCTCGGAGGTTGCCT	CTTGGAGC	183
*FGFR3*	NM_000142.5	F: TGCTGAAAGACGATGCCACTG R: CTTGCAGGTGTCGAAGGAGT	ACCTGCTG	224
*FGFR4*	NM_001291980.2	F: TTGCCAGCTTCCTACCTGAG R: GCTGGAGGTCAAGGAGTCAC	CTCTGCCT	110
*ACTB*	NM_001101.5	F: ATTGGCAATGAGCGGTTC R: GGATGCCACAGGACTCCA	CTTCCAGC	76

*ACTB*, gene that encodes β-actin; F, forward primer; *FGFR1-4*, genes that encode fibroblast growth factor receptors 1-4; *KL*, gene that encodes α-Klotho; R, reverse primer.

## Data Availability

Data is contained within the article. All the data can be provided upon request.
